# Regulation of output spike patterns by phasic inhibition in cerebellar granule cells

**DOI:** 10.3389/fncel.2014.00246

**Published:** 2014-08-25

**Authors:** Thierry R. Nieus, Lisa Mapelli, Egidio D'Angelo

**Affiliations:** ^1^Department of Neuroscience Brain Technology, Istituto Italiano di TecnologiaGenova, Italy; ^2^Neurophysiology Unit, Department of Brain and Behavioral Sciences, University of PaviaPavia, Italy; ^3^Neurophysiology, Brain Connectivity Center, C. Mondino National Neurological Institute, IRCCSPavia, Italy

**Keywords:** cerebellum, granule cell, GABA-A receptors, synaptic inhibition, modeling, spike timing

## Abstract

The complex interplay of multiple molecular mechanisms taking part to synaptic integration is hard to disentangle experimentally. Therefore, we developed a biologically realistic computational model based on the rich set of data characterizing the *cerebellar glomerulus microcircuit*. A specific issue was to determine the relative role of phasic and tonic inhibition in dynamically regulating granule cell firing, which has not been clarified yet. The model comprised the excitatory mossy fiber—granule cell and the inhibitory Golgi cell—granule cell synapses and accounted for vesicular release processes, neurotransmitter diffusion and activation of different receptor subtypes. Phasic inhibition was based on stochastic GABA release and spillover causing activation of two major classes of postsynaptic receptors, α1 and α6, while tonic inhibition was based on steady regulation of a Cl^−^ leakage. The glomerular microcircuit model was validated against experimental responses to mossy fiber bursts while metabotropic receptors were blocked. Simulations showed that *phasic inhibition* controlled the number of spikes during burst transmission but predicted that it specifically controlled time-related parameters (firing initiation and conclusion and first spike precision) when the relative phase of excitation and inhibition was changed. In all conditions, the overall impact of α6 was larger than that of α1 subunit-containing receptors. However, α1 receptors controlled granule cell responses in a narrow ±10 ms band while α6 receptors showed broader ±50 ms tuning. *Tonic inhibition* biased these effects without changing their nature substantially. These simulations imply that phasic inhibitory mechanisms can dynamically regulate output spike patterns, as well as calcium influx and NMDA currents, at the mossy fiber—granule cell relay of cerebellum without the intervention of tonic inhibition.

## Introduction

Synaptic inhibition controls local microcircuit functions by regulating membrane potential and calcium influx in neurons, with important consequences on spike generation and synaptic plasticity (Llinás et al., 2005; Mann and Paulsen, 2007; Lamsa et al., 2010; Wenner, 2011; Griffen and Maffei, 2014). Since neurons can tune their spike patterns on the millisecond scale (Eccles, 1973; Timmann et al., 1999), it is important to understand how the interplay of excitatory and inhibitory synapses regulates spike timing. Although recent works have raised attention toward the role of tonic inhibitory mechanisms, the role of phasic inhibition was less considered. This reflects the difficulty in dissecting the different components of inhibition pharmacologically and in activating excitatory and inhibitory fibers independently in arbitrary phase relationship.

A prototypical case is posed by the GABAergic inhibitory synapses made by Golgi cells on cerebellar granule cells (Eccles et al., 1964). Granule cells are normally silent at rest and are activated by the mossy fibers. The Golgi cells in turn are activated both by mossy fibers and granule cells (Cesana et al., 2013) setting up a double feed-back and feed-forward inhibitory circuit. The inhibitory process occurs in the cerebellar glomerulus, a specialized structure enwrapped into a glial sheet, in which several granule cell dendrites are activated by mossy fibers and inhibited by Golgi cells. The limited diffusion space in the glomerulus was shown to favor the establishment of tonic GABA levels and various effects of neurotransmitter spillover and cross-talk (Vos et al., 1999; Forti et al., 2006; Solinas et al., 2007a,b; Kanichay and Silver, 2008). Thus, two inhibitory mechanisms coexist in granule cells. *Tonic inhibition*, which was shown to control the granule spike number (Brickley et al., 1996) and transmission gain (Mitchell and Silver, 2003). *Phasic inhibition*, which was shown to limit the duration of granule cell responses (D'Angelo and De Zeeuw, 2009) and was also implicated in different functions requiring dynamic network control, including generation of granular layer coherent oscillations and resonance (Maex and De Schutter, 1998; Dugué et al., 2009; Solinas et al., 2010; Gandolfi et al., 2013), induction of mossy fiber - granule cell LTP and LTD (Mapelli and D'Angelo, 2007; D'Errico et al., 2009), and spatio-temporal reconfiguration of granular layer activity (Mitchell and Silver, 2003; Mapelli et al., 2010a). Phasic and tonic inhibition are based on different α1- and α6-subunit containing GABA-A receptors and on their differential localization and activation by direct release, spillover and tonic GABA levels (Nusser et al., 1995; Brickley et al., 1996; Rossi and Hamann, 1998; Hamann et al., 2002; Rossi et al., 2003; Farrant and Nusser, 2005; Mapelli et al., 2009, 2014). Although much emphasis has been given to the role of tonic inhibition in regulating granular layer functions, how these mechanisms dynamically regulate granule cell spike patterns has remained largely unexplored. This is somehow surprising for the granular layer, a neuronal microcircuit which is thought to provide dynamic regulation of spike timing (Eccles, 1973; Fujita, 1982; Timmann et al., 1999).

By exploiting the advanced knowledge on microstructural and molecular properties available, we have modeled the impact of *phasic* and *tonic* GABAergic inhibition on granule cell output spike patterns in response to coactivation of excitatory and inhibitory synapses by a short mossy fiber burst (Pellerin and Lamarre, 1997; Hartmann and Bower, 1998; Chadderton et al., 2004; Rancz et al., 2007; Arenz et al., 2008) and have predicted the impact of single-pulse activation of excitatory and inhibitory synapses with variable phase lag and arbitrary combinations of excitatory and inhibitory synapses (Pellerin and Lamarre, 1997; Hartmann and Bower, 1998; Chadderton et al., 2004; Rancz et al., 2007; Arenz et al., 2008). Model simulations revealed a critical and specific role for phasic inhibitory mechanisms in dynamic regulation of granule cell firing and calcium currents without the need of tonic inhibition.

## Methods

### Experimental methods

#### Whole-cell patch-clamp recordings

Patch-clamp recordings in acute cerebellar slices were performed as reported previously (D'Angelo et al., 1995; Armano et al., 2000; Mapelli et al., 2009). Briefly, 17- to 23-day-old Wistar rats were anesthetized with halothane (Aldrich, Milwaukee, WI) and killed by decapitation. Parasagittal 220 μm-thick acute slices were cut from the cerebellar vermis in cold Krebs solution and maintained at 30°C before being transferred to a 2-ml recording chamber mounted on the stage of an upright microscope. The preparations were perfused with Krebs solution (2 ml/min) and maintained at 30°C with a Peltier feedback device (TC-324B, Warner Instruments, Hamden, CT). Krebs solution for slice cutting and recovery contained (in mM): 120 NaCl, 2 KCl, 1.2 MgSO_4_, 26 NaHCO_3_, 1.2 KH_2_PO_4_, 2 CaCl_2_, and 11 glucose, and was equilibrated with 95% O_2_–5% CO_2_ (pH 7.4). The patch-clamp pipette solution contained (in mM): 81 Cs_2_SO_4_, 4 NaCl, 2 MgSO_4_, 0.02 CaCl_2_, 0.1 BAPTA, 15 glucose, 3 ATP-Mg, 0.1 GTP, and 15 HEPES. For experiments with current-clamp recording the internal solution contained (in mM): 126 potassium gluconate, 4 NaCl, 5 Hepes, 15 glucose, 1 MgSO_4_, 0.1 BAPTA-free, 0.05 BAPTA-Ca, 3 Mg^2+^-ATP, 0.1 Na^+^-GTP. These solutions maintained resting free [Ca^2+^] at 100 nM and pH was adjusted to 7.2. Patch-clamp pipettes filled with these solutions had a resistance of 5–8 MΩ before seal formation. Transient current analysis yielded a membrane capacitance (*Cm*) of 3.9 ± 0.3 pF, membrane resistance (*Rm*) of 2.0 ± 0.2 MΩ, and series resistance (*Rs*) of 17.1 ± 0.9 MΩ (*n* = 7). The 3-dB cell plus electrode cut-off frequency was *f*_VC_ = (2π R_s_C_m_)^−1^ = 2.5 ± 0.1 kHz (*n* = 7). All drugs were obtained from Sigma, except BAPTA tetrapotassium salt (Molecular Probes, Eugene, OR); GABA-B receptor blockers CGP35348 and CGP55845, mGluRs blockers MCPG, CPPG and gabazine (SR-95531, Tocris-Bioscience). All experiments were conducted in accordance with international guidelines from the European Community Council Directive 86/609/EEC on the ethical use of animals.

#### Experiments with metabotropic receptors blockers

**Figure 1:**
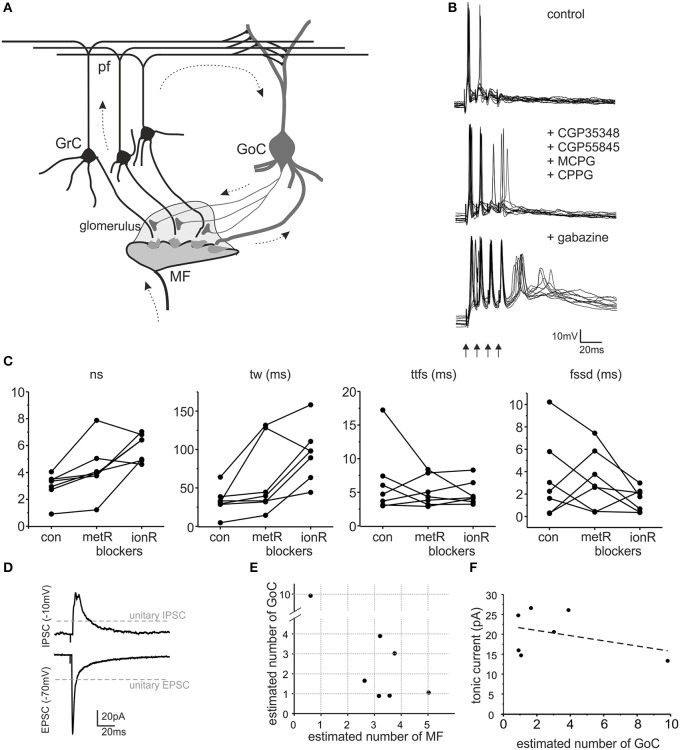
**The impact of GABA-A receptors on granule cell excitation**. **(A)** Schematic drawing of the cerebellar glomerulus. The granular layer of the cerebellar cortex receives input from mossy fibers (MF), making synaptic contacts with granule cells (GrC) and Golgi cells (GoC) in specialized structures called *glomeruli* (*light gray area*). GrC axons (parallel fibers - pf) contact GoC dendrites. Therefore, GoCs inhibit GrCs through a *feedforward* (MF-GoC-GrC) and a *feedback* (MF-GrC-GoC-GrC) pathway (*arrows*) (modified from Mapelli et al., 2014). **(B)** The traces show patch-clamp recordings of GrC activity following a brief burst of MF stimulation (100 Hz, *arrows*). Glutamatergic and GABAergic metabotropic receptor blockers (MCPG, CPPG, CGP35348, and CGP55845) modify GrC response. The subsequent blockage of ionotropic GABA-A receptors (gabazine) causes an increase in GrC excitability and spike generation. **(C)** The plots show the number of spikes (*ns*), time window (*tw*), time to first spike (*ttfs*) and first spike standard deviation (*fssd*) in control (*con)* and after metabotropic receptors blockage *(metR)* and GABAergic ionotropic receptor blockage *(ionR)* in 7 GrC recordings performed as illustrated in **(B)**. **(D)** Average EPSC and IPSC recorded from the same cell as in **(B)**. Note the different holding potential used to isolate the two currents. The gray lines indicate the amplitude of unitary EPSCs and unitary IPSCs. **(E)** The plot reports the estimated number of MF and GoC connections active in each one of the 7 experiments covered in this figure. **(F)** The plot shows that the tonic current measured after gabazine perfusion in each experiment does not correlate with the number of GoC connections (linear fitting slope −0.65, *R*^2^ = 0.137).

GABA-B receptor blockers CGP35348 and CGP55845, and the mGluRs blockers CPPG and MCPG were used at 100, 1, 300, and 500 μM, respectively. Perfusion of 10 μM gabazine further blocked the GABA-A receptor mediated inhibition. Incoming mossy fibers were stimulated with 4 pulses at 100 Hz, delivered every 10 s. Initially, no blockers were added to the external solution, the cells were maintained at the holding potential of −65 mV and the granule cells spikes in response to the stimuli were recorded. The same pattern was repeated 30–40 times in control, in presence of metabotropic receptors blockers and after perfusion of gabazine (added to the previous blockers, Figure 1B).

#### Tonic current estimates

The amount of tonic inhibition for each recording was estimated measuring the holding current change (Vh = −10 mV) after gabazine perfusion (on average 21.50 ± 2.12 pA *n* = 6, Figure 1F).

#### Neurotransmission parameter estimates

The specific synaptic organization of the cerebellar glomerulus enables a precise determination of synaptic parameters. EPSCs and IPSCs amplitude, recorded at Vh = −70 and −10 mV respectively, was used to estimate the number of excitatory and inhibitory synapses activated by the stimulus. The number of mossy fibers were estimated by the ratio between peak amplitude of EPSCs and single-fiber responses (sfEPSCs) recorded from granule cells in similar experimental conditions (Sola et al., 2004). With an average EPSC amplitude = −81.0 ± 12.9 pA (*n* = 7) and sfEPSC amplitude = −25.8 ± 3.3 pA, the EPSC/sfEPSC amplitude ratio yielded an average of 3.14 activated fibers per granule cell. The number of activated Golgi cell axons was estimated by the ratio between peak amplitude of evoked IPSCs (eIPSC) and spontaneous IPSCs (sIPSC) in the same granule cells. The sIPSCs depend on Golgi cell autorhythmic activity and are generated at single connections (Hamann et al., 2002; Rossi et al., 2003; Mapelli et al., 2009). On average, eIPSCs amplitude was 48.5 ± 17.8 pA, while sIPSCs amplitude was 16.3 ± 1.5 pA, so that eIPSC/sIPSC amplitude ration yielded 2.97 GoC axons activated per granule cell (see Mapelli et al., 2009). The parameter space explored in the model was based on the estimates of the number of input contacts (i.e., 1–4 MFs and 0–4 GoC fibers). The comparison between experimental data and model estimates therefore was shown for the 5 granule cells that showed up to 4 GoC and MF inputs (cf. Figure 1E, see **Figures 4, 5** and Table 1).

**Table 1:** **The table summarizes the number of excitatory (n MF) and inhibitory (n GoC) synapses activated during each experimental recording (exp 1–7)**.

	**n MF**	**n GoC**
exp1	3 (3.17)	1 (0.89)
exp2	1 (0.64)	10 (9.82)
exp3	3 (2.63)	2 (1.65)
exp4	4 (3.57)	1 (0.91)
exp5	3 (3.21)	4 (3.90)
exp6	4 (3.75)	3 (3.02)
exp7	5 (5.02)	1 (1.06)

*The number of excitatory synapses was estimated by comparing the average amplitude of eEPSCs with the unitary EPSC amplitude (reported in Sola et al., 2004), while the number of inhibitory synapses was estimated by comparing the average eIPSCs amplitude with the sIPSCs amplitude in these same recordings (see Methods). The estimated number of active synapses is indicated in brackets, while the unitary value was used to compare the experimental results to the model*.

### Modeling

In this paper, a computational model of neurotransmission and excitation in the cerebellar glomerulus is developed and a series of routines to investigate the parameter space is implemented. A single-compartment model of the granule cell [(D'Angelo et al., 2001) updated in Nieus et al. (2006)] was implemented with advanced representations of inhibitory synapses and run using the NEURON simulator (NEURON version 7.3; Hines and Carnevale, 2001). The model was coupled to a recording electrode to reproduce realistic current-clamp conditions and simulations were compared to cellular responses corrected for 10 mV liquid-junction potential. The ionic mechanism were the same as reported in Nieus et al. (2006) and Solinas et al. (2010). The granule cells are extremely compact also when channels are open in the dendrites during synaptic transmission. Calculations based on cable equation (D'Angelo et al., 1993, 1995; Rossi et al., 1994) and tests using bio-realistic compartmental models (Silver et al., 1992; Diwakar et al., 2009) showed that dendritic electrotonic length was around *L* = 0.04 and the maximum current and membrane potential loss was <5%. Therefore, the results would not be different using single or multi-compartment models.

#### Major aspects of the inhibitory mechanisms used as the base for modeling

In the cerebellar glomerulus, GABA released from the Golgi cell presynaptic terminal can act directly on receptors facing the postsynaptic site (fast direct component) or indirectly on extrasynaptic receptors through spillover from neighboring sites (slow indirect component) (Rossi and Hamann, 1998; Hamann et al., 2002; Rossi et al., 2003; Mapelli et al., 2009). Direct release from the presynaptic terminal activates α1 GABA-A receptors and, in part, α6 GABA-A receptors. While α1 receptors are located in the PSD, α6 receptors are located preferentially at extrasynaptic sites (but in part also in the PSD, Nusser et al., 1995). Fast kinetics of the direct component are explained by rapid gating of α1-containing GABA-A receptors. Conversely, slow kinetics of the indirect component are explained by slow gating of α6 receptors largely due to spillover from neighboring contacts (Rossi and Hamann, 1998; Hamann et al., 2002; Rossi et al., 2003). In addition, ambient GABA at sub-micromolar concentration activates high affinity α_6_ GABA-A receptors generating tonic inhibition.

#### Mathematical model of inhibitory synaptic transmission

A mathematical model of Golgi cell—granule cell neurotransmission was developed considering available knowledge on the synapse and on GABA-A receptor kinetics (Cherubini and Conti, 2001; Farrant and Nusser, 2005). The Golgi cell—granule cell IPSCs are generated by different GABA-A receptor subtypes: α_1_ subunit-containing receptors contribute mainly to early IPSC activation and determine the IPSC peak, while α_6_ subunit-containing receptors sustain the IPSC along its decay phase (Tia et al., 1996; Rossi and Hamann, 1998; Brickley et al., 2001). Differential receptor activation reflects a range of GABA affinities for the different subtypes. In particular, α_1_-subunit containingreceptors have EC_50_ in the 10–100 μM range, α_6_β_2/3_γ_2_ receptors have EC_50_ in the μM range, and α_6_β_2/3_δ receptors have EC_50_ in the 10 nM range (Tia et al., 1996; Nusser et al., 1998; Hadley and Amin, 2007). Differential receptor activation also reflects their subcellular localization. While α_1_ receptors are localized in the synaptic cleft, α_6_β_2/3_δ receptors are hundreds of nanometers away from the postsynaptic densities (Nusser et al., 1998), where they are most likely activated by low ambient GABA concentrations and by GABA spillover (10 nM to few μM, (Farrant and Nusser, 2005)). The α_6_β_2/3_γ_2_ receptors are localized in the synaptic junction (Nusser et al., 1998). The simulation of GABAergic neurotransmission in the glomerulus required explicit representations of GABA release and diffusion as well as of the kinetic properties of different GABA receptor subtypes.

***Mechanisms of eIPSC generation***. GABA release was implemented through a phenomenological 3-state model (ref. Markram and Tsodyks) that well reproduces synaptic dynamics on short time scale, as already demonstrated at the mossy-fiber to granule cell synapse (Nieus et al., 2006). The scheme was governed by recovery rates and release probability from the reserve pool and its output was GABA release. The neurotransmitter concentration, [GABA], was modeled by summing a pulse to a diffusive term:

(1) $$[\text{GABA}]={\left[\text{GABA}\right]}_{\text{P}}+{\left[\text{GABA}\right]}_{\text{D}}$$

**Figure 2:**
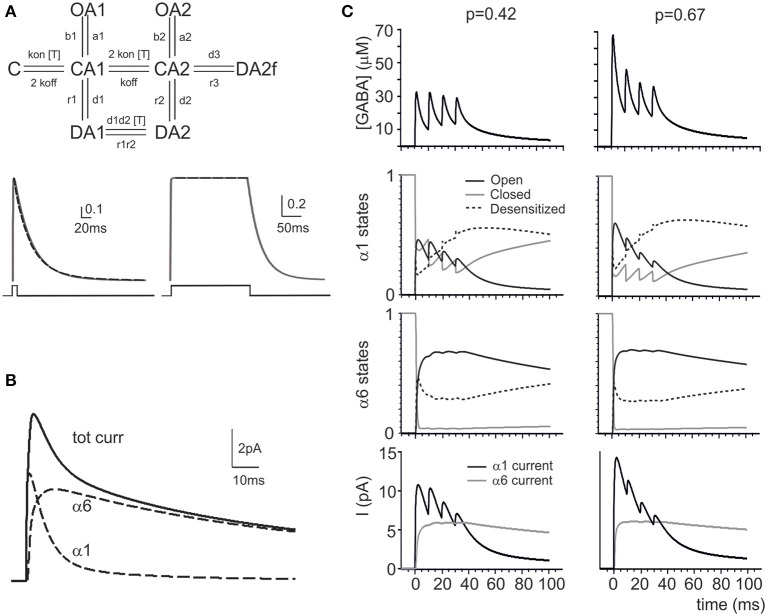
**Modeling GABA receptor-mediated responses in the cerebellar glomerulus**. **(A)** Schematic representation of the kinetic scheme for α_1_ and α_6_ subunit-containing receptors (modified from Pugh and Raman, 2005). The traces (*continuous lines*) show responses to brief and long 1 mM GABA pulses (taken from Tia et al., 1996). The simultaneous fitting of the decay phase of the α_6_ response to a fast (1 ms) GABA neurotransmitter pulse (1 mM) and of the almost constant response to a long (200 ms) GABA neurotransmitter pulse (1 mM) is shown (*dashed lines*) (see Methods and **Table 3** for details). **(B)** The simulated eIPSC generated by the model at 0 mV in response to a 1 ms GABA pulse shows amplitude and kinetics reproducing experimental eIPSCs. The contribution of the α_1_ and α_6_ components in the eIPSC is shown, revealing that the α_1_ receptors account for the direct response while the α_6_ receptors account for the indirect response (*dashed lines*). **(C)** The simulations show the impact of changing *pI* (from 0.42 to 0.64) on the GABAergic response to a train of impulses at 100 Hz. The *pI* increase changes the profile of GABA concentration (in particular, the first peak of GABA concentration is enhanced) modifying the kinetic states of α_1_ and α_6_ responses (O, C, and D indicate the sum of all the open, closed and desensitized states for each receptor type). The α_1_ is more affected than α_6_ response by *pI*, since α_6_ receptors reacts slowly and tends to saturate. The associated changes in the α_1_ and α_6_ currents are shown at the bottom.

[GABA]_P_ activates the receptors facing the releasing site, while [GABA]_D_ accounts for GABA spillover in the glomerulus (Rossi and Hamann, 1998; Nieus et al., 2006). Neurotransmitter concentration at a distance *r* from the releasing site was described by the diffusion equation:

(2) $${\left[\text{GABA}\right]}_{\text{D}}=\frac{M}{4h\pi D_{eff}}{\text{e}}^{\frac{{-\text{r}}^{2}}{{4\text{tD}}_{\text{eff}}}}$$

where *M* represents the amount of released neurotransmitter molecules, *D_eff_* is the effective diffusion coefficient of GABA molecules, *h* is the cleft thickness and *r* is the distance from which the neurotransmitter diffuses. Given the strong similarity of GABA and glutamate molecules (which differ only for a carbossilic group), the diffusion coefficient of GABA was set equal to that of glutamate (*D_eff_* = 0.221 μm^2^/ms; Nielsen et al., 2004; Nieus et al., 2006). From morphometry, *h* was set at 50 nm (Hámori and Somogyi, 1983; Jakab and Hámori, 1988). The parameters *M* and *r* in Equation 2 could not be estimated independently and were obtained from fitting (see below and Table 2). To account for the coexistence of direct and indirect components we implemented two separate GABA-A receptor kinetic schemes. The α_1_- and α6- receptor responses were reproduced using the same structural kinetic scheme (see Figure 2A) while the kinetic rates of the two receptor subtypes were determined by optimization to data taken from the literature (Figures 2A,B). The IPSC was then reproduced as:

(3) $$\text{IPSC}=\text{eIPSC}\alpha_{1}+\text{eIPSC}\alpha_{6}$$

**Table 2:** **Model parameters obtained from eIPSC train fittings with the model**.

	**Control**	**CGP55845**
*G^α1^_max_* (pS)	1323.25 ± 310.99	
*G^α6^_max_* (pS)	491.58 ± 112.32	
*A* (n°molec)	10573 ± 3324	
*r* (μm)	1.07 ± 0.54	
τ_*REC*_(ms)	38.7 ± 38.6	
τ_*FAC*_(ms)	0	
*p*	0.42 ± 0.08	0.67 ± 0.09

**Figure 3:**
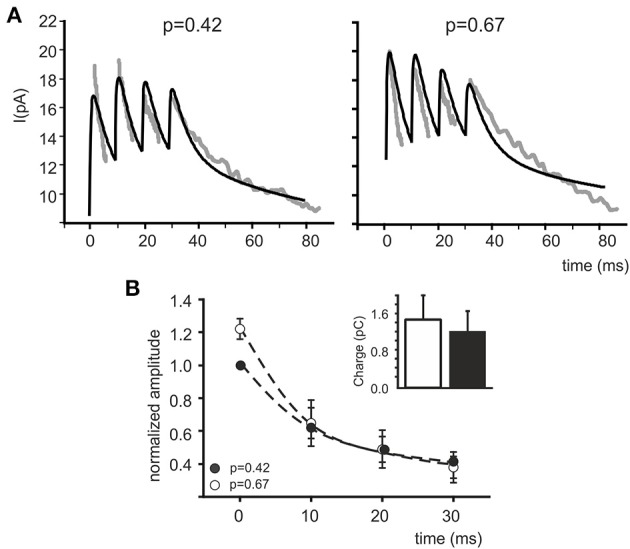
**Fitting eIPSC trains with the model**. **(A)** Fittings of experimental traces in control and after CGP55845 perfusion (taken from Mapelli et al., 2009). Both traces are fitted simultaneously with the model maintaining the same α_1_ and α_6_ receptor density and kinetic schemes, the same diffusion parameters and the same presynaptic (τ_R_, τ_F_) parameters. Only *pI* is left free to vary independently in the two traces. The fittings properly follow the experimental traces by adapting *pI* as the only independent parameter. **(B)** The plot shows normalized peak amplitude (mean ± MSE) obtained from fittings (*n* = 5) of eIPSC trains (*n* = 5). Similar to real data measurements (see Mapelli et al., 2009), the time course of changes after increasing *pI* demonstrates the increase in the first eIPSC and the tendency to attain control amplitude in subsequent responses (mean ± MSE). The histogram represents charge transfer during the simulated eIPSC trains, showing no significant difference between control and after increasing *pI* (*white bar* and *black bar*; *p* = 0.55 paired *t*-test).

G^α*1*^_max_ and G^α*6*^_max_ are the maximum conductance of α_*1*_ and α_*6*_ receptors, A is the number of diffusing molecules and r is the diffusion distance, τ_REC_ and τ_FAC_ are the time constants for synaptic vesicle recovery and facilitation, p is release probability. Note that since in all fits the facilitation time constant remained pretty low, τ_FAC_ was fixed to 0 without affecting the quality of the fit itself. The data, except for p, are in common since control and CGP55845 traces were fitted simultaneously and p was the only independent parameter. Data are reported as mean ± s.e.m. (n = 5 cells). The model eIPSC train fittings and states are shown in Figures 2, **3**.

The α_6_ component mostly sustains the eIPSC tail after the α_1_ response has almost completely decayed (cf. Wall, 2002). Diffusion also contributes to protract the eIPSC through the α_1_ component. The GABA channels were given a reversal potential V_Cl_ = −65 mV and the tonic component was modeled as a background conductance (D'Angelo et al., 2001).

***GABA-A receptor kinetics***. The GABA-A receptor models are characterized by two desensitized, three closed and two open states (Barberis et al., 2000; Jones et al., 2001). These models are based on IPSC reconstruction from out-side-out patch-clamp experiments. In whole cell in slices, IPSCs show faster decay than in patches, probably reflecting an increased desensitization caused by phosphorilation of the GABA receptor (Brickley et al., 2001). Interestingly, parallel investigations (Petrini et al., 2011) have demonstrated that the discrepancy between slice and out-side-out experiments might also be determined by the neurotransmitter diffusion in the synaptic cleft that can also shape the IPSC time course.

Recently, a multistate model was proposed that accounts for out-side-out and slice IPSC responses (Pugh and Raman, 2005). The Pugh-Raman model suggests that increasing the transition rate into the fast desensitizing state (“DA2f” in the scheme of Figure 2A) can convert out-side-out into slice channel behavior. The α_1_ receptor model was obtained by fitting the Pugh-Raman model to sIPSCs generated by the GABA pulse. The α_6_ receptor model was obtained by fitting the Pugh-Raman model to slow eIPSCs activated by GABA spillover (see Figure 2 in Tia et al., 1996; Rossi and Hamann, 1998; Wall, 2002). Fittings yielded an EC_50_ ≈ 1.5 μM placing the α_6_-model in the activation range for α_6_β_2/3_γ_2_ receptors. Then, α_1_ and α_6_ receptor currents combined to generate the composite eIPSC. The kinetic rate constants of α_1_ and α_6_ receptors are reported in Table 3.

**Table 3:** **Kinetic rate constants used to simulate α_1_ and α_6_ GABA-A subunit-containing receptors in cerebellar granule cells**.

	**α_1_**	**α_6_**
k_on_(mM^−1^· ms^−1^), k_off_ (ms^−1^)	20, 6	54.8, 0.31
a_1_ (ms^−1^), b_1_ (ms^−1^)	0.06, 0.03	0.06, 0.03
a_2_ (ms^−1^), b_2_ (ms^−1^)	0.4, 10	0.4, 10
d_1_ (ms^−1^), r_1_ (ms^−1^)	3.3e-4, 7e-4	0.86, 0.04
d_2_ (ms^−1^), r_2_ (ms^−1^)	1.2, 6e-3	2.7, 0.43
d_3_ (ms^−1^), r_3_ (ms^−1^)	15, 3.75	15, 7.41
d_1_d_2_ (mM^−1^· ms^−1^), r_1_r_2_ (ms^−1^)	15, 0.007	24.2, 0.09

The corresponding simulated traces and kinetic scheme are shown in Figure 2.

***Simulation methods***. The model was written in NEURON based on a previous reconstruction of the granule cell (for details see D'Angelo et al., 2001; Nieus et al., 2006) and is available on http://senselab.med.yale.edu/modeldb/default.asp. The model allowed to determine neurotransmission parameters through a fitting procedure carried out in two steps. First, single eIPSCs were fitted to determine GABA diffusion parameters (except for *h* and *D_eff_*, which were reported from literature). Then, the whole eIPSC train was fitted on data from our previous experimental investigation (Mapelli et al., 2009) yielding parameters related to synaptic dynamics including the vesicle recycling and facilitation time constants (τ_REC_ and τ_FAC_) and release probability (*p*).

***Stochastic model***. In order to test the GRC responsiveness in a more natural context we introduced synaptic stochastic mechanisms at the excitatory and inhibitory synapses. We modeled stochastic pre-synaptic release of the excitatory and inhibitory phasic components (i.e., the α_1_ receptor). The stochastic quantal release model was implemented as in Arleo et al. (2010) by splitting each synapse into a fixed number of independent releasing sites (RS) and independent post synaptic densities (PSD). The presynaptic RSs and the postsynaptic PSDs were endowed with the same kinetic scheme as in the deterministic model. The synaptic conductances were rescaled by the number of RSs. Each stimulus, e.g., presynaptic AP, is broadcast to the RSs and an uniform [0,1] random number (*rnd*) is generated at each RS where a fixed concentration of neurotransmitter (*Tmax*) is released when the corresponding *rnd* falls below the actual releasable resources (*Y*). This stochastic model yields the deterministic response when averaged over multiple trials (not shown).

#### Model of the glomerular microcircuit and simulations

In order to test the responsiveness of the granule cell (GC) in a realistic context, a GC microcircuit was built that included the model of the GC (D'Angelo et al., 2001) as well as its excitatory (Nieus et al., 2006) and inhibitory synapses (developed in this manuscript). At excitatory and inhibitory synapses three independent releasing sites were included (Sola et al., 2004; Mapelli et al., 2009; Arleo et al., 2010), each one comprising the same stochastic release mechanisms. The GC received a maximum of four excitatory mossy fibers (MF) and four inhibitory Golgi (GoC) axons. These average numbers were adopted to limit the parameters space and the case of higher numbers of synapses (e.g., 10 inhibitory synapses in Figure 1E) or releasing sites (e.g., see Sola et al., 2004; Saviane and Silver, 2007; Mapelli et al., 2009) were not accounted for.

**Figure 4:**
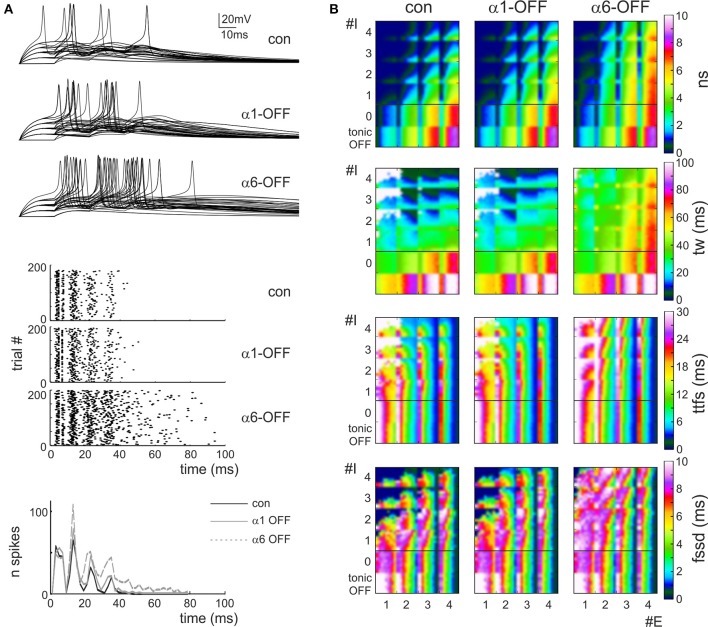
**Effect of the phasic and tonic inhibition on granule cell firing in response to mossy fiber bursts**. **(A)** Exemplar voltage traces simulated using *MF* = 2, *Pe* = 0.3, GoC = 2, Pi = 0.2 [cf. coordinates (2.3, 2.2) in the *E/I* space in **B**]. The traces show responses in control and after switch-off of α_1_ and α_6_ subunit containing receptors. **(B)** *E/I* response space for number of spikes (*sn)*, time window (*tw)*, time to first spike (*ttfs*), first spike standard deviation (*fssd*) in control (con) and after switch-off α_1_ and α_6_ subunit containing receptors (α1-OFF and α6-OFF) and of the GABA tonic current (tonic OFF). The *E/I* space is generated by reporting the number of active excitatory (1 < *E* < 4) and inhibitory (0 < *I* < 4) synapses on the two axes and by using colors to represent the parameter values. For each E/I combination, a subquadrant identifies a *pE/pI* subspace (0 < pE < 1 on the x-axis, 0 < pI < 1 on the y-axis). Note that α_6_ subunit-containing receptors are most effective in modifying the parameters.

**Figure 5:**
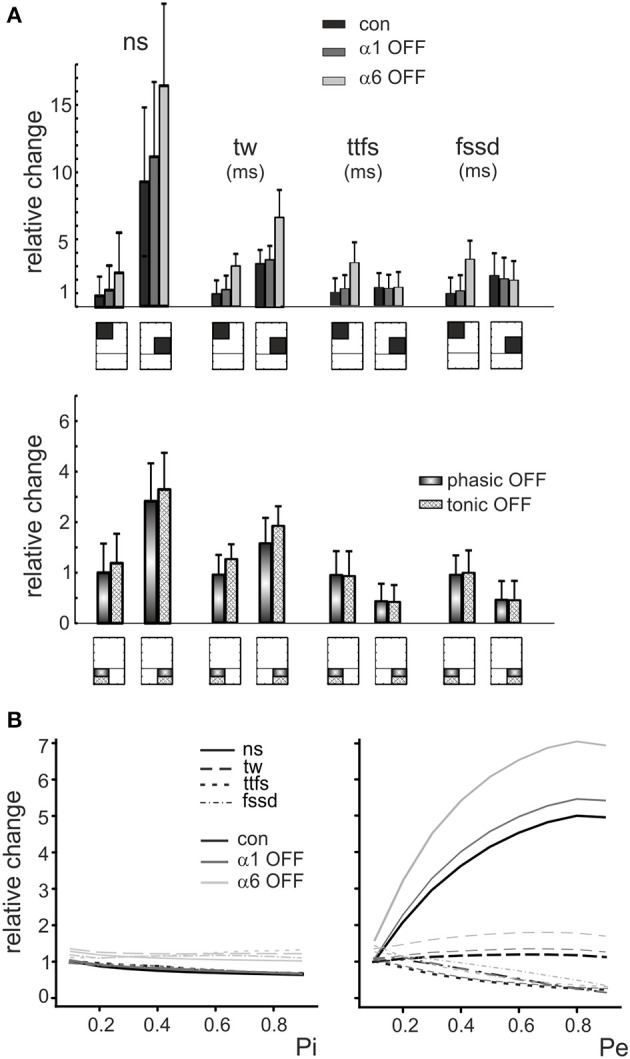
**The role of E/I and pE/pI in controlling granule cell firing parameters in response to mossy fiber bursts. (A)** (Top) The relative changes of number of spikes (*ns*), time window (*tw*), time to first spike (*ttfs*) and first spike standard deviation (*fssd*) in control (con, *black*) and after switch off of α1 (α1-OFF, *gray*) or α6 (α6-OFF, *light gray*) receptors is shown for the low-*E/I* and high-*E/I* quadrants identified in Figure 4B. It should be noted that the *E/I* balance primarily affects *ns* and *tw*.(*Bottom*) The relative change of number of spikes (*ns*), time window (*tw*), time to first spike (*ttfs*) and first spike standard deviation (*fssd*) is compared with phasic inhibition off (*phasic OFF*) and tonic inhibition off (*tonic OFF*). Blocking tonic inhibition does not influence notably the effect of phasic inhibition on the time-related spiking parameters, time to first spike and first spike standard deviation. **(B)** The relative changes of number of spikes (*ns*), time window (*tw*), time to first spike (*ttfs*) and first spike standard deviation (*fssd*) are reported as a function of the inhibitory (and excitatory release probability (*Pi, Pe*), showing that *Pi* affects modestly the parameters, while raising Pe remarkably increases number of spikes and decreases time to first spike and first spike standard deviation.

**Figure 6:**
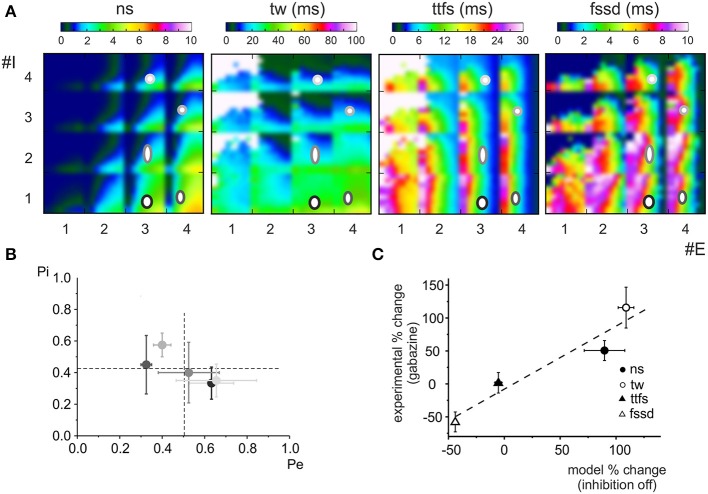
**Model predictions of experimental GABA-A receptor blockage**. **(A)** The experimental parameters reported in Figures 1C,E are reported on the control *E/I* response space for the 5 cells showing *E* < 4 and *I* < 4 yielding the corresponding *Pe* and *Pi* values. The position of symbols and their size correspond to the mean ± MSE of data values extrapolated from number of spikes (*ns*), time window (*tw*), time to first spike (*ttfs*) and first spike standard deviation (*fssd*) (same gray scale as in **B**). **(B)** The *Pi* and *Pe* values obtained in A and averaged across the different parameters (*sn, tw, ttfs*, and *fssd*) are reported as mean ± MSE for each of the 5 cells considered in **(A)**. The release probabilities felt in a narrow range around the average values of *Pe* = 0.51 ± 0.06 and *Pi* = 0.42 ± 0.04 (dashed lines). **(C)** The plot compares the relative change of number of spikes (*ns*), time window (*tw*), time to first spike (*ttfs*) and first spike standard deviation (*fssd*) after gabazine perfusion in the 5 recordings shown in **(A,B)** (mean ± MSE) with that caused by switching off phasic and tonic inhibition in the model. The data points were fitted with a linear function yielding a slope of 0.95 (*R*^2^ = 0.914, *n* = 4).

**Figure 7:**
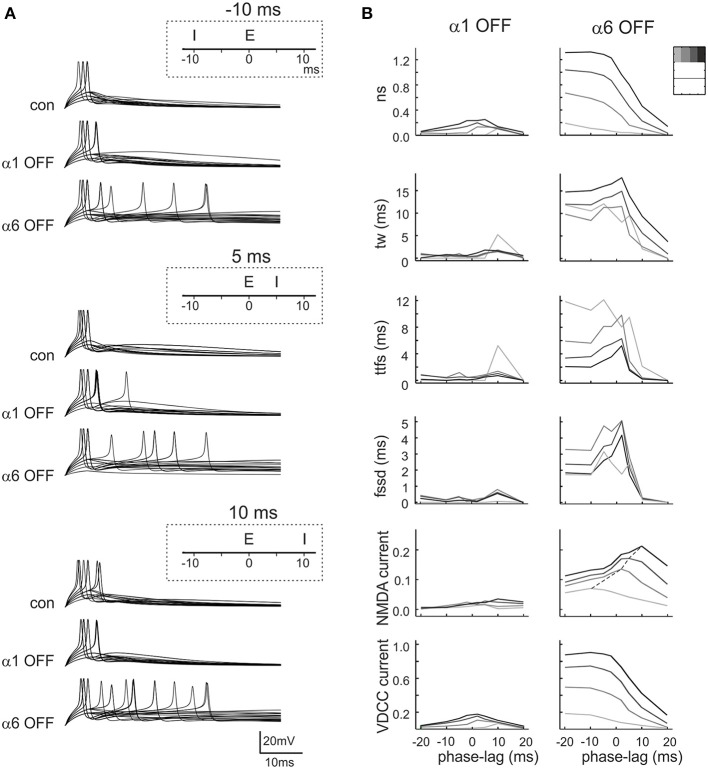
**Effect of phasic inhibition on granule cell response with variable E/I phase**. **(A)** Exemplar simulations of inhibition occurring at different phases compared to excitation (E/I phase lag of −10, 5, 10 ms, as indicated in the insets) in control and after α1 or α6 receptor switch-off. **(B)** The plots show the changes of number of spikes (*ns*), time window (*tw*), time to first spike (*ttfs*), first spike standard deviation (*fssd*), VDCC currents and NMDA currents occurring at different E/I phase lags. The data represent the differences (α1_OFF_-control) and (α6_OFF_-control) for simulations carried out with 3–4 inhibitory fibers and 1–4 excitatory fibers (gray coded as indicated in the inset). Each line is the interpolation to average data obtained with different release probabilities. It should be noted that the data distributions show resonance around 0 ms.

**Figure 8:**
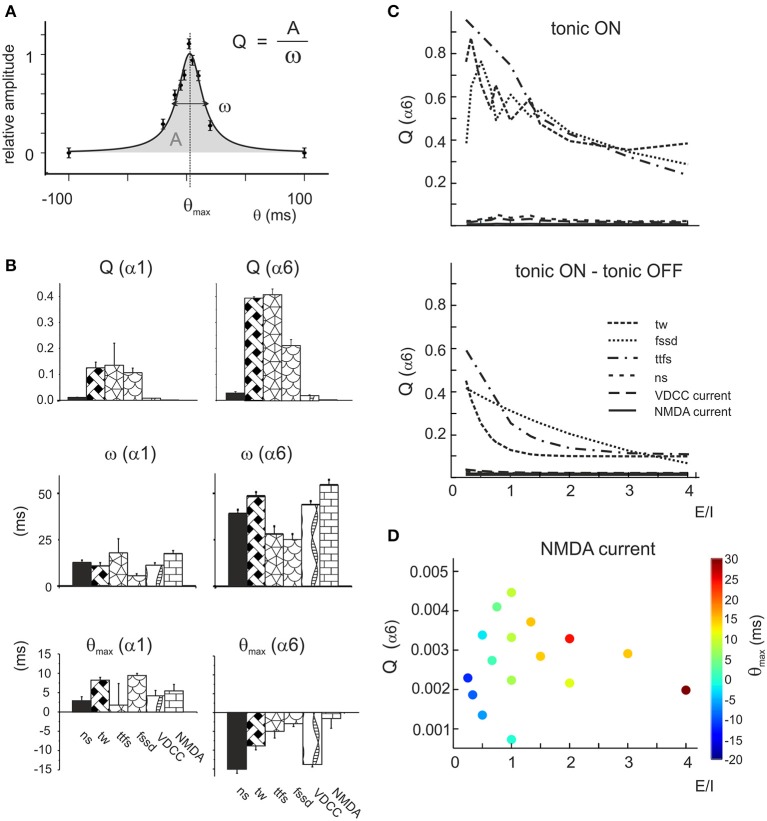
**Resonance of the parameter distributions at different E/I levels. (A)** Exemplar fitting with a Lorentzian function (Equation 4) applied to the phase-lag plot (cf. Figure 7B) indicating the geometrical meaning of the parameters *Q, A*, ω, *θ_max_*. **(B)** The average values of Lorentzian parameters during α1 or α6 switch-off are reported for number of spikes (*ns*), time window (*tw*), time to first spike (*ttfs*), first spike standard deviation (*fssd*), VDCC current and NMDA current. Note that *Q*(α6) and ω(α6) is always larger than *Q*(α1) and ω(α1), while *θ_max_*(α6) is negative and *θ_max_*(α1) is positive. These data are averaged over the whole parameter response space. **(C)** The *top* panel shows the *Q* factors of the α6_OFF_ difference phase-lag curves (α6_OFF_-CTRL). Note that *Q* of time window (*tw*), time to first spike (*ttfs*) and first spike standard deviation (*fssd*) decrease with increasing *E/I*, while the other parameters are much less affected. The b*ottom* panel shows the difference *Q* factors obtained as *Q* (tonic OFF)–*Q* (tonic ON). Note that the trends of these Q plots is qualitatively similar to that reported above. **(D)** Precession of *θ_max_*(α6) for the NMDA *Q* factor. The phase of the peak (color coded) increases with increasing *E/I*.

Two stimulation protocols were used to simulate the impact of the different inhibitory mechanisms on the granule cell output. First, a high frequency input (100 Hz, 4 pulses) was used to activate the excitatory synapses, while the inhibitory synapses were activated by the same input train delayed by 5 ms to mimic synaptic and integration delays in the feed-forward inhibitory MF-GoC-GC pathway. The results obtained with the first protocol are reported in **Figures 4–6**. Secondly, single shock stimuli were delivered to the excitatory and inhibitory synapses with a variable phase-lag (from −100 to +100 ms). The results obtained with the second protocol are reported in **Figures 7, 8**.

To the ease of comparison with experimental results we monitored set of parameters such as the number of output spikes (*sn*), the timing of the first spike (*ttfs*), the first spike standard deviation (*fssd*, defined as the standard deviation of the measure of *ttfs*) and the time window of the output spikes (*tw*, defined as the time difference between the last spike and the first stimulation). Reliable estimates of the measured parameters were then obtained by running 500 runs for each tested condition.

#### Data analysis

All data were analyzed with custom codes developed in Python (www.python.com) based on the Scipy (www.scipy.com), Numpy (www.numpy.com) and MatPlotLib (www.matplotlib.com) modules. The analysis allowed to quantify the number of spikes elicited, the time window, the time of the first spike and its variability (i.e., first spike precision) in the tested *E/I* conditions. In a set of simulations we also recorded the Calcium and NMDA current traces and computed the mean values over a 130 ms long time window.

A compact representation of the *E/I* space (e.g., **Figure 4B**) was obtained by reporting the excitatory and inhibitory strengths on Cartesian coordinates using a color-code map, in which a color scale corresponded to the intensity of the measured parameter. The excitatory, inhibitory strengths (*S_E_, S_I_*) were quantified as the sum of the active fibers (MF/GoC) and their release probability (*Pe*/*Pi*), such that *S_E_* = MF + *Pe* and *S_I_* = GoC + *Pi*.

The *S_E/I_* was calculated on sub-regions of the *E/I* space by averaging over the active fibers involved and the corresponding release probabilities (e.g., **Figure 4B**). Each sub-region was named after the number of active fibers involved: for example, *E/I*(1/1) corresponds to 1 excitatory and 1 inhibitory fiber. *S_E/I_* yields a comprehensive quantification of the changes of the measured quantities at variable *E/I* levels.

In order to describe the shape of *S_E/I_* data distributions at variable phase-lag between synaptic excitation and inhibition, the data were fitted with a Lorentz equation *y(θ)* of the form (Gandolfi et al., 2013):

(4) $$y\left(\theta \right)=\frac{2\cdot A}{\pi }\frac{\omega }{4\cdot \left(\theta -\theta_{max}\right)2+\omega^{2}}$$

where, *A* is the underlying area, *θ_max_* is the peak frequency, and ω is the width at *y(θ_max_*)/2. Lorentzian fittings allowed to find the resonance phases (*RF* = *θ_max_*) and to calculate *RA* = *y(θ_max_*)/ω, which corresponds to the quality factor *Q* in resonance literature and characterizes a resonator's bandwidth relative to its center delay. The goodness of fit was assessed by calculating the squared correlation factor, *R*^2^. *Q* is normally used in signal processing theory and describes the tendency of a signal to show resonance vs. monotonic roll-off.

The *E/I* response space representation also distinguishes between different settings of the GABA tonic current when the phasic component is zero.

#### Entropy calculation

To estimate the redundancy of the parameters (*sn, tw, ttfs*) on the *E/I* space we calculated the coding fraction (Borst and Theunissen, 1999; Sadeghi et al., 2007) defined as:

(5) $$CF_{P}=\frac{MI_{P}}{H(S)}$$

where *H(S)* is the entropy of the *E/I* input space and *MI* quantifies the mutual information of the measured parameters *P* (*P* = *sn, tw, ttfs*). Each *S_E/I_* configuration of the *E/I* space was regarded as an independent stimulus. The cardinality of the stimuli is therefore given by: 4 (MF) × 9 (*Pe*) × 5 (GoC) × 9 (*Pi*) = 1620. *CF_P_* measures the fraction of *S_E/I_* that can be distinguished based on the observation of the whole output generated by the model (i.e., 500 trials for each configuration) and is normalized between 0 (no separation, 100% redundant) and 1 (perfect separation, 0% redundant). *CF* calculation was performed with the Python module “*pyentropy*” (Ince et al., 2009) that provides appropriate procedures to robustly estimate *MI*.

## Results

In this paper we have developed a computational model of the cerebellar glomerular microcircuit (Figure 1A) and we have simulated its functionality exploring the response space governed by synaptic inhibition. Simulation results were compared with experimental responses to mossy fiber burst stimulation.

### The effect of inhibitory neurotransmission on granule cell responses to mossy fiber bursts

Short high-frequency bursts represent a fundamental pattern of mossy fiber activity (Chadderton et al., 2004). Bursts co-activate granule cells and Golgi cells (D'Angelo et al., 1995; Mapelli et al., 2014). While granule cells depolarization begins, the progressive increase of activity in the inhibitory circuit tends to modulate their response. An early component of inhibition passes through the mossy fiber—Golgi cell—granule cell circuit (feed-forward inhibition), while a late component passes through the mossy fiber—granule cell—Golgi cell—granule cell circuit (feed-back inhibition) (Cesana et al., 2013; D'Angelo et al., 2013).

The impact of inhibitory neurotransmission was investigated by measuring how it modulated granule cell excitation following mossy fiber burst stimulation in acute cerebellar slices (a condition in which feed-forward inhibition prevails). Since metabotropic mechanisms can influence granule cell responses (see (Mapelli et al., 2014) for review), ionotropic effects were isolated using a cocktail of antagonists for mGluRs and GABA-B receptors (CGP35348, CGP55845, MCPG, CPPG) (Figure 1B). Following application of these blockers, on average the number of spikes increased (*sn* change 39.8 ± 10.5%, *p* < 0.034, *n* = 7), the discharge time-window increased (*tw* change 93.4 ± 43.1% *p* < 0.034, *n* = 7) while the time-to-first-spike and first spike standard deviation did not vary significantly (*ttfs* change −2.3 ± 17.5%, *n* = 7, *p* = 0.79; *fssd* change −1.17 ± 1.34%, *n* = 7, *p* = 0.97) (Figure 1C). These results show that metabotropic receptors could modify burst transmission to granule cells indicating that the data to be compared to our cerebellar microcircuit model simulations had to be those obtained in the presence of metabotropic receptor blockers.

Inhibitory neurotransmission between Golgi cells and granule cells is mediated by GABA-A receptors, which can be selectively blocked by the GABA-A receptor antagonist, gabazine (Rossi et al., 2003). This receptor antagonist is normally used in a variety of experimental conditions and blocks the phasic and tonic inhibitory processes altogether (Hamann et al., 2002; Nusser and Mody, 2002; Mitchell and Silver, 2003; Rossi et al., 2003; Mapelli et al., 2010a,b; Gandolfi et al., 2013). Following metabotropic receptor blockage (see above), the application of 10 μM gabazine significantly increased number of spikes (68.9 ± 41.1%, *p* < 0.05, *n* = 7) and time window (112.6 ± 45.0%, *p* < 0.04, *n* = 7) but did not significantly change the time to first spike (2.74 ± 10.83%, *p* < 0.79, *n* = 7) or first spike standard deviation (23.4 ± 80.0%, *p* < 0.32, *n* = 7) (Figure 1C). Thus, as a whole, GABA-A receptor-mediated inhibition reduced the intensity and duration of granule cell responses without significantly changing the average time of occurrence and precision of the first spike. This was expected considering that Golgi cell inhibition through the feed-forward circuit usually occurs after the granule cell has emitted the first spike.

In each of the recorded granule cells, measurements of EPSCs and IPSCs were obtained at the reversal potential of IPSCs and EPSCs, respectively (Figure 1D). Moreover, the tonic GABA current was estimated by measuring the leakage current before and after gabazine application (not shown). We did not found any significant correlations between the tonic current and the estimated number of active Golgi cell axons (Figure 1F), suggesting that the tonic current was regulated by ambient GABA rather than by phasic neurotransmitter release (for review see Mapelli et al., 2014). Specific simulations showed that changes in the tonic current in the range observed experimentally did not significantly affect the model outcome (<5% difference for spike number, first-spike delay, time-window; data not shown). Therefore, in the model tonic inhibition was set as a fixed value, not correlated with the number of active synapses.

The number of active synapses was estimated by comparing EPSCs and IPSCs with the average single-site responses measured previously in identical experimental conditions (Figure 1D) (Sola et al., 2004; Mapelli et al., 2009). The estimated number of active mossy fibers ranged from 1 to 5 and that of active Golgi cell synapses ranged from 1 to 10 (Figure 1E). Therefore, for each recorded granule cell, the most relevant parameters required to determine its inhibitory control were available and this allowed a direct comparison with simulated data (see **Figure 6**).

### Modeling inhibitory neurotransmission

A model of cerebellar glomerular inhibition should be able to predict the different kinds of synaptic inhibitory responses observed experimentally in granule cells.

#### mIPSCs

The minimal GABAergic responses are the miniature synaptic currents, or *mIPSCs*, which correspond to release of single neurotransmitter quanta (Mapelli et al., 2009).

#### sIPSCs

The spontaneous multiquantal GABAergic responses, or *sIPSCs*, are caused by spontaneous activity in Golgi cells (Dieudonne, 1998; Rossi and Hamann, 1998; Forti et al., 2006; Solinas et al., 2007a,b; Mapelli et al., 2009; Brandalise et al., 2012; Hull and Regehr, 2012). Since *sIPSCs* reflect activation at single Golgi connections, *sIPSCs* are examples of *direct* release without spillover from neighboring contacts.

#### eIPSCs

The evoked IPSCs elicited by electrical stimulation of the Golgi cell axon, or *eIPSCs* (Rossi and Hamann, 1998; Mapelli et al., 2009), are composed by both a *direct component* due to release of GABA from the presynaptic site facing the postsynaptic density and an *indirect component* due to spillover of neurotransmitter from neighboring presynaptic sites. The decay of *eIPSCs* is indeed slower than that of *sIPSCs*.

Inhibitory synaptic transmission in the cerebellar glomerulus was reconstructed considering the following salient aspects (see Methods for details): (i) the vesicular release cycle contained an explicit representation of the number of releasing sites and of release probability, (ii) the mechanism of vesicle fusion was stochastic, (iii) the postsynaptic membrane expressed α1 and α6 receptors, (iv) GABA diffusion was explicitly modeled, and (v) tonic GABA receptor activation was accounted for.

The granule cell GABA receptor models were characterized by two desensitized, three closed and two open states (Barberis et al., 2000; Jones et al., 2001) whose transitions were differentially controlled by GABA transients and slow diffusion waves (Petrini et al., 2011). The GABA receptor responses were modeled using a modified Pugh and Raman (2005) 8-state kinetic scheme using the parameters optimized to match responses generated by GABA pulses (α_1_-type; Brickley et al., 2001) and GABA spillover (α_6_-type; Tia et al., 1996; Rossi and Hamann, 1998; Wall, 2002) (see Methods for details; Figure 2A; Tables 2, 3). The *eIPSCs* were reconstructed as the sum of α_1_ and α_6_ receptor-mediated components (Figure 2B). The time course of α_1_ and α_6_ receptor-mediated currents and their combinations were compatible with the time course of *sIPSCs* and *eIPSCs* (Mapelli et al., 2009).

The response of the model during repetitive synaptic transmission is shown in Figure 2C. The *eIPSC* peak showed depression along the train, while the *eIPSC* tail showed the marked temporal summation typical of experimental recordings. Clearly, subsequent *IPSC* peaks were driven by oscillations in the activation state of α_1_ receptors, while temporal summation was mostly determined by integration of GABA signals through α_6_ receptors. The α_6_ receptors activated slowly and, due to their high affinity, tended to saturate in a few impulses. Interestingly, changing release probability had a remarkable impact on α_1_ receptor-mediated peaks but much less so on the α_6_ receptor-mediated current plateau, as further considered below.

### Modeling the effect of release probability changes

A raise in release probability was proposed to explain the changes in synaptic responses caused by GABA-B receptor blockage by CGP55845 (Mapelli et al., 2009). Here, these changes were simulated by increasing the release probability from *pI* = 0.42 to *pI* = 0.67 (Figure 2C). This transiently enhanced GABA concentration in the synaptic cleft, but within 4 impulses the concentration settled toward similar levels with both *p* values. While the α_1_ receptors followed the GABA concentration changes, the α_6_ receptors developed similar kinetics at both release probabilities. The slow reaction of α_6_ receptors and their early saturation explained the invariance of temporal summation and total charge transfer despite the clear changes caused by *pI* on the α_1_ receptor-mediated response.

In order to test whether a *p* change could indeed provide a global interpretation of IPSC changes during trains, control and CGP55845 eIPSC trains reported by Mapelli et al. (2009) were simultaneously fitted with the model and *pI* was left free to vary (Figure 3A). The fittings yielded *pI* = 0.42 ± 0.12 in control and *pI* = 0.67 ± 0.13 in CGP55845 (*n* = 5), in close agreement with experimental determinations (*n* = 5; *p* = 0.14, paired *t*-test). The normalized eIPSC amplitude showed a significant difference on the first pulse but then recovered to control values and the total electrical charge transferred during the IPSC train was in good agreement with the experimental one and did not change significantly by raising *pI* (from 1.19 ± 0.20 to 1.46 ± 0.24 pC; *n* = 5; *p* = 0.31 paired *t*-test) (Figure 3B). These simulations indicate that a raise in release probability can indeed explain a consistent set of changes in composite α_1_–α_6_ receptors-mediated synaptic currents during repetitive neurotransmission suggesting appropriate setting of synaptic transmission parameters.

### Modeling the effect of inhibition on granule cell firing

In order to quantify granule cell responsiveness in different activation regimens, several series of simulations were run using the *dendritic microcircuit model* (Figure 4A) and the spiking parameters (number of spikes, time-window, time to first spike and first spike standard deviation) were calculated from raster plots and PSTHs of the responses (see Methods). An exhaustive exploration over all possible excitatory/inhibitory (*E/I*) synapse combinations and release probabilities was performed generating multiparametric color plots (Figure 4B).

Some regularities emerged from this representation. First of all, the parameters varied in a predictable manner with respect to the *E/I* balance: number of spikes and time window apparently increased by several times while time to first spike and first spike standard deviation showed modest changes by raising *E/I*. On a macro-scale, in all graphs an ideal bisectrix separated a low response region (top left, low *E/I*) from a high response region (bottom right, high *E/I*). Moreover, a micro-pattern emerged inside each sub-quadrant determined at each *E/I* by varying excitatory and inhibitory release probabilities, *pE*/*pI*. Thus, in principle, by combining different numbers of fibers with different release probabilities, a broad set of response combinations could be obtained generating an extended response space. A quantitative insight on its properties was obtained by considering representative areas. For example, by comparing parameters in the quadrants E1,2/I3,4 (*low E/I*) and E3,4/I1,2 (*high E/I*) there was a nearly 10-fold increase in number of spikes, a 3-fold increase in time window and a modest changes in time to first spike and first spike standard deviation (Figure 5A).

The inspection of the graphs reported in Figure 4B showed that, after switch-off of the phasic components of inhibitory synaptic transmission, number of spikes and time window increased in all quadrants, while time to first spike and first spike standard deviation increased only at low *E/I*. Interestingly, the effect of α6-receptor switch-off dominated over that of α1-receptor switch-off in all cases. It should also be noted that the effects of changing *E/I* and *pE*/*pI*, as well as the effects of α1 and α6-receptors switch-off, tuned first-spike timing and precision over the sub-millisecond time-scale and that the spike number varied in discrete units of a few spikes, making the system highly sensitive to activity variations. The complete switch-off of all forms (phasic and tonic) of GABAergic inhibition (simulating a gabazine application see Figure 1) caused a proportionate increase only in spike number and time-window duration without changing time to first spike and first spike standard deviation.

Finally, the model showed that raising *pI* caused a modest decrease (≤20%) of number of spikes, time window, time to first spike and first spike standard deviation. Conversely, raising *pE* remarkably increased number of spikes (≤80%) and decreased time to first spike and first spike standard deviation (≤80%) with a limited effect on time window (≤20%) (Figure 5B). Therefore, the effect of synaptic inhibition on burst parameters were more related to the number of active inhibitory synapses and to *E/I* than to the inhibitory release probability.

### Experimental data in the excitatory/inhibitory space

The simulated parameters were compared to those measured experimentally (cf. Figure 1). The points were reported in their sub-quadrant following the indications derived from Figure 1E and located at the corresponding *pE*/*pI* intersection (Figure 6A). In this comparison we used only the 5 granule cells with *E, I* = 4 in order to fit into the simulated parameter space. For these cells, the *pE* and *pI* values closely corresponded to those reported previously (Sola et al., 2004; Mapelli et al., 2009) (Figure 6B). The parameter variation caused by gabazine was then compared to that caused by switch-off of both tonic and phasic synaptic inhibition in the model (Figure 6C). The data measured for the different parameters showed a variation similar to that of the model (linear fitting with slope 0.95, *R*^2^ = 0.914). It should be noted that this data distribution was not significantly different from the unitary diagonal (*X*^2^ *p* < 2.6 *e*^−10^). Finally, it should be noted that time to first spike and first spike standard deviation showed modest changes compared to number of spikes and time window, in agreement with experimental results matched for the E/I balance (experimental *E/I* = 2.2 compared to simulated *E/I* = 2.6). Therefore, the model was able to capture the main quantitative properties of glomerular transmission and the impact of synaptic inhibition on granule cell firing.

### Information coding in the excitatory/inhibitory space

In order to assess the impact of inhibition on information coding (i.e., to what extent the same responses could be obtained with different parameters), the coding fraction (actually the ratio between mutual information and entropy, see Methods) was calculated (Borst and Theunissen, 1999; Sadeghi et al., 2007; Ince et al., 2009). The coding fraction of number of spikes, time window, and time to first spike was relatively small (see Table 4) demonstrating a remarkable redundancy in the parameter space. Once inhibition was removed by blocking α1 and α6 receptors, the coding fraction increased for time window and time to first spike, but not apparently so for the number of spikes (Table 4), indicating a preferential control of inhibition over spike patterning and timing rather than on spike number *per se*.

**Table 4:** ***Coding fraction* of number of spikes (*sn)*, time window (*tw)* and time to first spike (*ttfs)* was relatively small demonstrating a remarkable redundancy in the parameter space**.

	**sn**	**tw**	**ttfs**
CTRL	0.123	0.144	0.192
α1OFF	0.128	0.165	0.211
α6OFF	0.121	0.181	0.233

*Once inhibition was removed by blocking α1 and α6 subunit-containing receptors, the coding fraction increased for time window and time to first spike, but not apparently so for the number of spikes, indicating a preferential control of inhibition over spike patterning and timing rather than on spike number per se*.

### The impact of E/I phase on spike generation and on NMDA and VDCC currents

Beside the coordinated action of excitation and inhibition caused by concurrent mossy fiber activation of granule cells and Golgi cells shown in Figure 4, the excitatory and inhibitory synapses impinging on granule cells can work in any arbitrary phase relationship. Given their different time-course and temporal integration during trains (cf. Figures 2, 3), α1 and α6 receptor-mediated currents might have differential impact on firing depending on the *E/I* phase (Figure 7A). Therefore, we have performed simulations using different phase-lags of inhibition with respect to excitation generating *tuning curves* for the spiking parameters (in these simulations *pE* and *pI* were not changed since we used single stimulation pulses preventing their impact on short-term train dynamics). The most apparent changes occurred when α6 receptors were switched off. Interestingly, number of spikes, time window, time to first spike and first spike standard deviation showed resonance around −10 ms phase-lag. When α1 receptors were switched off, resonance appeared around 10 ms phase-lag. The impact of the *E/I* phase was evaluated also for currents generated by NMDA channels and voltage-dependent calcium channels (VDCCs) (Figure 7B). Again, the most apparent changes occurred when the α6 receptor was turned off. The VDCC current decreased monotonically with positive phase-lag while the NMDA current showed broad tuning distributed between −20 and +30 ms phase-lag. When the α1 receptor was turned off, resonance peaked around +10 ms phase-lag.

These simulations showed that α1 and α6 receptor-mediated effects combined in tuning spike generation toward specific phase-lags and that α6 receptor-mediated effects were larger and broader than α1 receptor-mediated effects.

### Modulation of tuning curves by the E/I phase and tonic inhibition

The tuning curves reported in Figure 7 revealed dependence of granule cell responses on the *E/I* phase-lag. This effect was evaluated by fitting the tuning curves with Lorentzian functions yielding *Q* = *A/ω*, a parameter indicating the tendency of the response to peak at a certain phase-lag *θ_max_* with area *A* and broadness ω (Equation 4, see Methods; Figure 8A).

Lorentzian parameters, which report the effectiveness of tuning toward a preferential phase-lag, showed specific properties (Figure 8B). For all parameters, *Q*(α6) > *Q*(α1) reflected the larger impact of α6 than α1 receptor-mediated charge transfer, ω(α6) > ω(α1) reflected the longer duration of α6 than α1 receptor-mediated responses, and *θ_max_*(α6) < 0 and *θ_max_*(α1) > 0 reflected the slower raise of α6 than α1 receptor-mediated responses (cf. Figures 2, 3). Interestingly, *Q*(α6) was much larger for time window, time to first spike and first spike standard deviation than for number of spikes (Figure 8B). This pattern was opposite to that observed for burst transmission (cf. Figure 5A), implying that only the time-dependent parameters were tuned by the *E/I* phase difference. It should also be noted that *Q*(α6) of the NMDA current was very small, reflecting the slow time course of this current and its broad integration times (D'Angelo et al., 1995; Schwartz et al., 2012).

Modulation of *Q*(α6) for time window, time to first spike and first spike standard deviation tended to decrease with *E/I*, according to the powerful effect of phasic inhibition in controlling time-dependent parameters (Figure 8C). Modulation of *Q*(α6) with *E/I* for the VDCC current followed strictly that of the number of spikes (D'Angelo et al., 1999; Gandolfi et al., 2014). Modulation of *Q*(α6) with *E/I* for the NMDA current was marginal but, specifically, *θ_max_* (α6) shifted progressively from −20 to +30 ms (phase-lag precession, cf. Figure 7B) with increasing *E/I* (Figure 8D) unveiling a complex influence of α6 receptor-mediated inhibition on voltage-dependent NMDA channel unblock.

Finally, in order to determine the effect of tonic inhibition on granule cell responses to dynamic patterns, tuning curves were generated with tonic inhibition blocked. The plot in Figure 8C shows the difference between the cases in which tonic inhibition was on or off. The tonic current biased the effect of inhibition proportionately to the *E/I* balance without altering the dynamics of inhibition substantially. Therefore, the model predicts that tonic inhibition simply scales the effect of phasic inhibition on firing parameters.

## Discussion

In this paper we have developed a detailed *glomerular microcircuit model* in order to assess how phasic and tonic components of GABA-A receptor-mediated inhibitory neurotransmission contribute to dynamic regulation of firing in cerebellar granule cells. The main prediction of this model is that *phasic inhibition* can dynamically regulate granule cell firing in response to temporally organized patterns of mossy fiber and Golgi cell activity. Phasic inhibition preferentially controlled the number of spikes during transmission of short bursts, but it showed improved control over time-related parameters (time to first spike, first spike standard deviation and time window) when the *E/I* phase was changed. In all conditions, the overall impact of α6 was larger than that of α1 subunit-containing receptors. However, α1 receptors controlled granule cell responses in a narrow ±10 ms band while α6 receptors showed broader ±50 ms tuning. Eventually, phasic inhibition fine-tuned the initiation and termination of granule cell firing during burst-burst transmission, as well as the probability of firing at different *E/I* phases, on the millisecond-scale. The model also predicts that *tonic inhibition* can bias these effects without changing their nature substantially. These results imply that the specific organization of phasic inhibitory mechanisms can effectively regulate the dynamics of spike generation at the mossy fiber - granule cell relay of cerebellum without the intervention of tonic inhibition.

### The predictive power of the glomerular microcircuit model

The *glomerular microcircuit model* could fit and explain the firing patterns observed in experimental data following mossy fiber burst stimulation. Once experimental granule cell firing parameter (time to first spike, first spike standard deviation, number of spikes and time window) values were reported on the simulated response space, the *glomerular microcircuit model* yielded *pE* and *pI* values corresponding to those observed experimentally (average *pE* = 0.51 vs. 0.55 in (Sola et al., 2004); average *pI* = 0.42 vs. 0.42 in Mapelli et al., 2009). Moreover, the model properly fitted the spiking parameters changes caused by gabazine, a drug that blocks both phasic and tonic inhibition. Therefore the model was endowed with mechanisms capable of capturing the impact of inhibitory neurotransmission on granule cell firing parameters and could be used to predict the effect of different E/I phase lags.

It should be noted that in our model the effect of metabotropic receptors was excluded to isolate the ionotropic component of inhibition. Experimentally, the application of mGluRs and GABA-B receptor blockers caused a net dis-inhibitory effect (cf. Figure 1), which could reflect multiple mechanisms. The mGluRs on presynaptic Golgi cell terminals might have reduced GABA release, and this would suffice to explain the increased number of spikes and duration of discharge in most of the recordings. Conversely, GABA-B receptor blockage on granule cells and on Golgi cell terminals (Rossi et al., 2006; Mapelli et al., 2009) would reduce granule cell excitability and enhance inhibitory neurotransmission, thereby sorting the opposite effect. Future models of glomerula neurotransmission may be designed to include metabotropic receptor control (Mapelli et al., 2014).

### Properties of the inhibitory neurotransmission model

The inhibitory neurotransmission model was based on GABA-A receptor kinetics and was fitted to voltage-clamp recordings. Simulations of glomerular inhibition were consistent with a mechanism in which α_1_ receptors determine the rapid response to released GABA characterizing the direct component, while α_6_ receptors determine the slow temporal integration of spillover currents characterizing the indirect component (Cherubini and Conti, 2001; Farrant and Nusser, 2005; Glykys and Mody, 2007). The model predicts that, while α_1_ receptor-mediated responses can precisely follow GABA concentration changes, α_6_ receptor-mediated responses tend to saturate early during the train. Thus, as observed experimentally, raise in *p* determined minor changes in synaptic charge transfer matching experimental observations (Mapelli et al., 2009).

The adherence of the inhibitory neurotransmission model to the microscopic properties of the synapse could be defined at different levels. First, the GABA-A receptor kinetic schemes (derived from Pugh and Raman, 2005) appropriately reproduced eIPSCs and their changes in different functional conditions, suggesting that GABA affinities and the balance between open, closed and desensitized states were in the physiological range for granule cell receptors. Secondly, by setting the same diffusion coefficient for GABA and glutamate (DiGregorio et al., 2002), the number of diffusing molecules and the diffusion distance were similar to those estimated for the mossy fiber—granule cell synapse (Nieus et al., 2006). This was not unexpected, given the substantial similarity in the number of releasing sites involved and the common diffusion space for the two neurotransmitters. Thirdly, the *p* values obtained by fitting the model to experimental data were almost identical to those estimated experimentally with quantal analysis and no changes in the rate of vesicle recycling or in any other model parameters were needed to fit the experimental eIPSC trains.

### Inhibitory control of granule cell firing during burst transmission

Model simulations explained the complex effect of phasic inhibition on the burst-burst transmission modality of the granular layer (Chadderton et al., 2004; Rancz et al., 2007; Arenz et al., 2008). The *E/I* balance was most effective in controlling granule cell tonic discharge (the ~9-fold number of spikes increase was associated with a ~3-fold time window increase, so that the output spike frequency increased by about 3 times) but had minor effects on time-related parameters, time to first spike and first spike standard deviation. The *pE/pI* balance was most effective in controlling number of spikes, time to first spike and first spike standard deviation. Raising *pE* specifically increased number of spikes and reduced time to first spike, consistent with the fact that AMPA and NMDA receptor activation shows a remarkable modulation with *pE* (Nieus et al., 2006). Raising *pI* depressed all parameters but its effect was small, probably because inhibition during a burst largely depends on the charge carried by α6 receptors, which are early saturated (see above and cf. Figures 2, 3). The first spike standard deviation was almost equally reduced by raising *pE* and *pI*, probably reflecting reduction of synaptic noise determined by the larger number of released quanta (Sola et al., 2004; Mapelli et al., 2009). It turns out that, during burst transmission, the major effect of inhibition is to control the number and frequency of spikes emitted by granule cells through a regulation of the relative number of active Golgi cell synapses. Both *E/I* and *pE/pI* effects were enhanced by GABA receptor switch-off, but the contribution of α6 was always much larger than that of α1 receptors reflecting the fact that α6 receptors controlled most of the inhibitory charge transfer.

The tonic GABA leakage in the model did not contribute to the effect of inhibitory transmission substantially beside proportionately increasing number of spikes and time window. The tonic GABA leakage was modeled on the basis of experimental observations in cerebellar slices. The cerebellar glomerulus is an ideal structure to preserve tonic inhibition in slices provided that the glial sheath is intact (Hamann et al., 2002; Mitchell and Silver, 2003; Mapelli et al., 2014). Although the topic is still debated, tonic inhibition was reported *in vivo* revealing properties similar as in slices (Chadderton et al., 2004), suggesting that predictions made by our model could apply to synaptic transmission in natural conditions.

### Inhibitory control of granule cell firing with variable E/I phase

Model simulations predicted a specific role for phasic inhibition in controlling granule cell responses with different E/I phases lags. The most apparent regulatory effects on granule cell firing occurred when the α6 receptor-mediated current was turned off. This was due to the long time course and large charge transfer of α6 receptor-mediated responses, extending the influence of inhibition for about ±50 ms around granule cell excitation. When the α1 receptor-mediated current was turned off, the effects were smaller and resonance was restricted over about ±10 ms. Interestingly, time-related firing parameters (time window, time to first spike, first spike standard deviation) showed much sharper tuning than tonic firing (number of spikes). Moreover, tuning of time-related parameters decreased with *E/I* along with the decreasing inhibitory control, while GABA leakage did not alter the dynamics of inhibition substantially.

The model also predicted how VDCC and NMDA currents would be regulated by α1 and α6 receptors. VDCC current regulation strictly followed the number of spikes, as these channels open during the upstroke (D'Angelo et al., 1997, 2001; Gandolfi et al., 2014). The α6 receptor-mediated regulation of the NMDA current showed a broad tuning extending over ±50 ms and reflecting the slow NMDA current kinetics (D'Angelo et al., 1995; Schwartz et al., 2012). Puzzlingly, the effect of inhibition on the NMDA current showed a precession with *E/I* (from −20 to +30 ms phase-lag) suggesting a complex voltage-dependent interaction between NMDA channel unblock, depolarization and inhibitory control.

### Implications for dynamic inhibitory control during circuit activity

The dynamic control of inhibition by α6 and α1 receptors could have specific consequences during local microcircuit activation. *Feed-forward* inhibition affects granule cell excitation around 5 ms after mossy fiber activation (D'Angelo and De Zeeuw, 2009). With 5 ms phase-lag, the model predicted a higher impact for α6 mediated inhibition (except for the number of spikes at low E/I, where α6 and α1 affected the response in a similar way). *Feed-back* inhibition occurs after about 10 ms. With 10 ms phase-lag, the overall effect was also dominated by α6 mediated inhibition, but α1 and α6 effects were similar for time-to-first spike and first-spike standard deviation (and also on the number of spikes at low E/I). Therefore, in summary, the α6 component dominated on feed-forward inhibition, while the α1 component gained relevance in controlling the timing of granule cell output firing at low E/I during feed-back inhibition. Interestingly, during feedback inhibition at low E/I the α1 component is often more effective than the α6 component. Concerning the NMDA current, phase-lag precession suggested that the maximum impact of α6 mediated inhibition at 5–10 ms phase-lag was also achieved at low E/I. The multiple combinations of these effects and the complex inhibitory patterns provided by α1 and α6 subunit-containing GABA-A receptors make phasic inhibition particularly suitable to finely regulate the delay and precision of granule cell firing.

These properties may have an impact on spike-timing dependent plasticity (STDP) induction and on its interaction with low-frequency coherent oscillations reported in the cerebellar granular layer (Pellerin and Lamarre, 1997; Hartmann and Bower, 1998) and deserve future experimental assessment.

### Considerations on inhibitory control of information transfer

The extensive simulation strategy adopted in this paper suggests how phasic inhibition provided by Golgi cells controls information transfer through the granular layer. For each *E/I* value, release probabilities generated a complex subspace, such that similar firing parameter values could be found with different *E/I* and *pE/pI* combinations with a redundancy of 80 ÷ 90% (*CF* = 0.1 ÷ 0.2) (Borst and Theunissen, 1999; Sadeghi et al., 2007; Ince et al., 2009). The actual number of excitatory synapses may be relatively unimportant, provided that the number of inhibitory synapses is properly set, suggesting that the granular layer has intrinsic re-scaling properties with respect to the intensity of the inputs. Synaptic plasticity, mostly through *pE*, could allow to select points in this computational landscape for any specific *E/I* and *pE/pI* combination.

The amount of transmitted information (*MI*; Borst and Theunissen, 1999; Sadeghi et al., 2007; Ince et al., 2009), depends on the signal-to-noise (*S/N*) ratio. Since short bursts (1–3 spikes: see Figure 4B) at the mossy fiber—granule cell synapse carry the largest fraction of *MI* (Arleo et al., 2010), it is expected that regulation of first-spike delay and precision by phasic inhibition contributes substantially to regulate *MI* transfer through the cerebellum input stage (Garrido et al., 2013). It should also be noted that quantal fluctuations in GABA release generate synaptic noise, which should be smaller the larger the number of active synapses and the higher their release probabilities. Therefore, redundant *E/I* and *pE/pI* combinations giving same granule cell output patterns may not be equivalent in terms of *MI* transfer.

### The five reasons why phasic inhibition is needed for dynamic control

In summary, simulations identify five main reasons why phasic inhibition is needed for dynamic control of spike transmission at the mossy fiber—granule cell relay. (1) During burst transmission, tonic inhibition simply scales the effect of phasic inhibition on tonic spike discharge (*number of spikes*) without affecting time-related parameters (time to first spike and time window) significantly. (2) During inhibition with variable E/I phase, the pattern observed during bursts is reversed, so that phasic inhibition specifically affects time-related parameters without affecting tonic spike discharge significantly. (3) During inhibition with variable E/I phase, tonic GABA leakage does not alter the dynamics of inhibition substantially, so that tonic inhibition simply scales the effect of phasic inhibition on firing parameters. (4) The differential kinetics imposed by α1 and α6 receptors during inhibition with bursts or with variable E/I phase may not be explained by tonic inhibitory effects. (5) Phasic inhibition reduced redundancy specifically for time-related parameters.

## Conclusions

Biologically realistic simulations show that phasic inhibition, by exploiting the different kinetics of α1 and α6 receptor-mediated responses, can effectively regulate the delay and precision of granule cell firing. The effect of α6 receptors dominated during burst transmission while that of α1 receptors emerged during single-pulse transmission with variable phase lag, so that α1 and α6 receptors proved suitable for single-spike and burst control, respectively. These mechanisms in turn may regulate coincidence detection of calcium signals required for STDP and for its integration during theta-cycles (D'Angelo and De Zeeuw, 2009). Conversely, no specific effects of tonic inhibition occurred beyond a proportionate scaling of granule cell discharge duration and frequency, compatible with a gain control mechanisms over tonic firing (Mitchell and Silver, 2003). Therefore, these model simulations imply that phasic inhibition endows the cerebellar cortex with a mechanism suitable for improving spatio-temporal reconfiguration of the mossy fiber input and for promoting dynamic signal processing in the cerebellar cortex (Marr, 1969; Eccles, 1973; Fujita, 1982). Critical tests to this prediction could be obtained by experiments, in which multiple different mossy fiber and Golgi cell axon fibers are activated independently with arbitrary phases. Although this protocol would be impractical with current electrophysiology, it may become at hand by combining recently developed imaging and optogenetic techniques (e.g., see Gandolfi et al., 2014).

### Conflict of interest statement

The authors declare that the research was conducted in the absence of any commercial or financial relationships that could be construed as a potential conflict of interest.
