# Corrigendum: Regulation of output spike patterns by phasic inhibition in cerebellar granule cells

**DOI:** 10.3389/fncel.2016.00030

**Published:** 2016-02-16

**Authors:** Thierry R. Nieus, Lisa Mapelli, Egidio D'Angelo

**Affiliations:** ^1^Department of Neuroscience Brain Technology, Istituto Italiano di TecnologiaGenova, Italy; ^2^Neurophysiology Unit, Department of Brain and Behavioral Sciences, University of PaviaPavia, Italy; ^3^Neurophysiology, Brain Connectivity Center, C. Mondino National Neurological Institute, IRCCSPavia, Italy

**Keywords:** cerebellum, granule cell, GABA-A receptors, synaptic inhibition, modeling, spike timing

The corrigendum is needed in the acknowledgments section, since a funding agency was erroneously forgotten. We request therefore to add the sentence:

**.… and by grants of the Italian Ministry of Health (RF-2009-1475845) to ED**.

So that the full corrected paragraph will become:

## Acknowledgments

We thank Leda Roggeri for technical assistance. This work was supported by European Union grants [CEREBNET FP7-ITN238686, REALNET FP7-ICT270434, Human Brain Project (HBP-604102)] **and by grants of the Italian Ministry of Health (RF-2009-1475845) to ED**.

We confirm that missing information does not affect the scientific validity of the results at any extent.

## Author contributions

All authors listed, have made substantial, direct and intellectual contribution to the work, and approved it for publication.

### Conflict of interest statement

The authors declare that the research was conducted in the absence of any commercial or financial relationships that could be construed as a potential conflict of interest.

